# Foodborne Diseases in the Edible Insect Industry in Europe—New Challenges and Old Problems

**DOI:** 10.3390/foods12040770

**Published:** 2023-02-10

**Authors:** Remigiusz Gałęcki, Tadeusz Bakuła, Janusz Gołaszewski

**Affiliations:** 1 Department of Veterinary Prevention and Feed Hygiene, Faculty of Veterinary Medicine, University of Warmia and Mazury in Olsztyn, 10-719 Olsztyn, Poland; 2 Center for Bioeconomy and Renewable Energies, Department of Genetics, Plant Breeding and Bioresource Engineering, Faculty of Agriculture and Forestry, University of Warmia and Mazury in Olsztyn, 10-719 Olsztyn, Poland

**Keywords:** foodborne pathogens, entomophagy, biosecurity, microbiological safety, risk analysis, food chain

## Abstract

Insects play a key role in European agroecosystems. Insects provide important ecosystem services and make a significant contribution to the food chain, sustainable agriculture, the farm-to-fork (F2F) strategy, and the European Green Deal. Edible insects are regarded as a sustainable alternative to livestock, but their microbiological safety for consumers has not yet been fully clarified. The aim of this article is to describe the role of edible insects in the F2F approach, to discuss the latest veterinary guidelines concerning consumption of insect-based foods, and to analyze the biological, chemical, and physical hazards associated with edible insect farming and processing. Five groups of biological risk factors, ten groups of chemical risk factors, and thirteen groups of physical risks factors have been identified and divided into sub-groups. The presented risk maps can facilitate identification of potential threats, such as foodborne pathogens in various insect species and insect-based foods. Ensuring safety of insect-based foods, including effective control of foodborne diseases, will be a significant milestone on the path to maintaining a sustainable food chain in line with the F2F strategy and EU policies. Edible insects constitute a new category of farmed animals and a novel link in the food chain, but their production poses the same problems and challenges that are encountered in conventional livestock rearing and meat production.

## 1. Introduction

Insects (class Insecta) are ubiquitous in the world [1], and they come into direct contact with humans [2,3]. Social attitudes toward insects vary. In some countries, insects are regarded as ectoparasites and pests. However, in some cultures and ethnic groups, insects, as a source of protein and other nutrients, have been a part of the human and livestock diet for many centuries [4]. Many insect species are also used in traditional medicine around the world [5]. Insects are used in production of vaccines and protein preparations [6]. In 2004, extracts from *Lucilia sericata* larvae became the first insect-based treatment for chronic wounds that has been approved for use in the United States [7]. The venom of the Samsum ant (*Pseudomyrmex* sp.) has numerous medicinal properties. This powerful antioxidant has been shown to reduce inflammation, relieve pain, inhibit tumor growth, protect the liver, and aid hepatitis treatment [8,9]. Insects are also farmed animals [10]. Honey bees (*Apis mellifera*) have been exploited for honey for many millennia, whereas domestic silk moths (*Bombyx mori*) and Chinese oak silk moths (*Antheraea pernyi*) have long been reared for silk. Insects are also in human and animal diets.

Entomophagy, namely the practice of eating insects, continues to attract the interest of researchers, ecologists, and consumers as a potential solution to feeding the world’s growing population in the coming decades [11,12]. In recent years, insects have emerged as one of the most innovative substrates in human and animal nutrition [13,14]. According to many scientists, edible insects are a major milestone in efforts aiming to diversify protein sources and guarantee global food security [15]. Edible insects are most widely consumed in subtropical and tropical regions, but entomophagy is not highly popular in Western culture [11]. Global insect consumption is difficult to estimate, but, according to the literature, around 2000 insect species are consumed in more than 80 countries [16,17]. The most widely consumed insects belong to the orders Coleoptera (31% of global consumption), Diptera (2%), Hemiptera (10%), Hymenoptera (14%), Isoptera (3%), Lepidoptera (18%), Odonata (3%), and Orthoptera (13%) [18]. Around 1500 species of wild and farmed edible insects are eaten in Africa [19]. Nearly 96 tons of edible insects are consumed in the Democratic Republic of Congo each year, and, in Kinshasa alone, an average family consumes around 300 caterpillars per week [20]. Latin America is the second largest market of edible insects, and entomophagy is most popular in Brazil, Ecuador, Colombia, Mexico, Peru, and Venezuela [21]. The Asian insect market is highly innovative [22]. In Asia, insects are not only popular substrates in food and feed production but are also used in the pharmaceutical industry [22]. Until recently, edible insects had not been regarded as a major food source in Europe. A breakthrough came on 20 December 2017, when a list of novel foods, including insects, was introduced by Commission Implementing Regulation (EU) 2017/2470 [23].

All arguments in favor and against entomophagy should be considered to promote introduction of long-term sustainable solutions on the European food market. Safety of edible insects should also be thoroughly analyzed before these products are authorized for human, companion animal, and livestock consumption. Numerous guidelines have been developed to ensure that edible insects are reared under safe conditions and can be safely used in food and feed production [24,25,26]. Despite the fact that most species of edible insects are harvested without proper biosecurity from the natural environment [27], farmed insects have to meet additional food safety standards and guidelines, including control of foodborne pathogens [28,29,30]. For this reason, microbiological safety of edible insects has to be thoroughly researched before they are approved for mass production. The optimal parameters for insect rearing, processing, and storage have already been described in the relevant regulations, but many edible insect species have not been tested for microbiological safety. Edible insects can be a source of biological hazards, including bacteria that cause foodborne diseases, and insect-based foods can become contaminated in all stages of production, delivery, and consumption. Other biological risks associated with insect farming, such as use of organic side-streams and food wastes in insect nutrition, are often disregarded.

The aim of this article was to: (i) discuss the role of edible insects in the farm-to-fork (F2F) strategy, (ii) present current veterinary guidelines relating to safe use of edible insects in food and feed production, and (iii) analyze biological, chemical, and physical risk factors in edible insect farming.

## 2. Edible Insects in the Farm-to-Fork Strategy

According to the Food and Agriculture Organization (FAO) of the United Nations, daily protein consumption per capita will reach around 54 g in 2030 and 57 g in 2050 [31]. Daily protein consumption per capita increased from 39 g in 1961 to 52 g in 2011. The global protein supply will have to increase by 76% to cater to the growing demand [32]. The rapid increase in protein demand can be attributed not only to global population growth but also to higher daily protein intake. It is estimated that around 30% of the world’s land surface is used for cultivation of crops, whereas 7% of land is used for livestock production [11,33]. According to many researchers, livestock production has a significant impact on the environment by contributing to soil degradation, global warming, loss of biodiversity, greenhouse gas emissions, and air and water pollution [34]. These problems accentuate the need for a more sustainable approach to agricultural production. Many countries have committed to become carbon-neutral by 2050 as part of the European Green Deal and the F2F strategy. According to the United Nations (UN), the global population will reach 8.5 billion in 2030 and 9.7 billion in 2050, which implies that the transition to carbon neutrality will be a highly challenging process [35]. Rapid population growth will increase demand for food, but it will also decrease availability of land for agricultural production [33,36]. Livestock production is one of the most rapidly growing agricultural sectors, and increased demand for animal-based products will also drive demand for feed. However, availability of feedstuffs on the global market could be compromised in the current geopolitical climate. Complete and balanced diets are essential for maintaining animal health and performance. Livestock diets should be characterized by high protein content (*Hermetia illucens* and *Tenebrio molitor* meal contains 40–60% protein), high palatability and digestibility (*H. illucens* and *T. molitor* meal digestibility has been estimated at 91–95%), an optimal amino acid profile (*H. illucens* and *T. molitor* meal contains more threonine, valine, isoleucine, leucine, and lysine than fish meal), and fatty acid profile [37,38,39]. Feeds should be free of antinutritional factors and pathogens, and they should be thoroughly tested to eliminate health risks for animals and ensure food chain safety [40]. High-quality ingredients should be used in feed production to maximize livestock performance. At present, fish meal and soybean meal are the main sources of protein in animal diets [41,42]. Fish meal is produced mainly from fish species that have high bone and fat content and are not suitable for direct human consumption. Fish meal is an excellent source of protein, minerals, and vitamins; it has a favorable composition of amino acids and fatty acids and is highly digestible [40,42]. However, overfishing, the environmental impact of fisheries, and legal regulations have reduced profits in the fish meal industry and have decreased the supply of fish meal for feed production [43,44,45]. Genetically modified (GM) soybeans dominate on the global market, and they are one of the leading sources of protein in food and feed production [41,46]. At present, soybean production meets the current demand for protein. In 2014, GM soybeans were cultivated on 82% of land under soybeans and on 50% of land under genetically engineered crops worldwide. According to estimates, 93–95% of soybean meal on the global market comes from GM plants [47,48,49,50,51]. As a result, industrial livestock production, particularly in Europe and North America, is highly dependent on feeds containing GM soybeans [3]. In Europe, soybean yields are low due to the harsh climate, and the EU is the world’s second largest importer of feed protein. The EU imported 26 million tons of soybean meal and 15.9 million tons of soybeans in 2019 [47]. Innovative feed ingredients of comparable quality and profitability are being sought as part of the European Green Deal to minimize the EU’s dependence on soybean imports. Various alternative protein sources have been considered, including distiller-dried grains with solubles, rapeseed meal, and legume seeds (lupin seeds and fava beans). However, these ingredients must be tested for protein content, nutritional value, and presence of antinutritional factors to ensure high productivity and profitability. To maintain continuity of feed production, feed ingredients characterized by uniform quality and composition should be available on the market.

In recent years, insects have emerged as a viable alternative in food and feed production. According to research, edible insects can replace or supplement other high-protein feed components. The experiences of cultures that practice entomophagy suggest that insect farming has considerable potential for improving food security and that edible insects can be farmed on an industrial scale. Research shows that *H. illucens*, *Musca domestica*, *T. molitor*, and fish proteins have similar amino acid compositions [48,49]. According to the UN, entomophagy could help to reduce world hunger. Insects are a sustainable and environmentally friendly source of protein for animals and humans [35].

Despite the fact that entomophagy is a controversial or even shocking practice for many Western consumers [50,51], insects could substantially contribute to global food security in the future [52]. In the EU, several insect species’ protein has been approved for use in fish, poultry and pig feed, and pet food [10]. Several edible insect species, including *Acheta domesticus, Locusta migratoria,* and *T. molitor*, have also been approved for human consumption [10]. Insect farming is one of the most rapidly growing agricultural sectors [53]. In terms of volume, the edible insect market is projected to increase from 2000 tons in 2018 to around 200,000 tons in 2020 and 1.2 million tons in 2025 [54]. Insects make up a large part of diets consumed by wild animals [55], and insect protein is an important link in the food chain of many fish and poultry species under organic and natural conditions [56]. Insect-based feeds deliver health benefits and improve livestock welfare [57]. Edible insects are abundant in high-quality protein, and some insect species contain bioactive compounds with proven health benefits, including a beneficial amount of chitin (aids digestion), lauric acid (immunomodulatory properties), and antimicrobial peptides (bactericidal properties) [58]. In Europe, some insect farms cater specifically to the needs of the pet food industry. Numerous scientific and commercial initiatives suggest that popularity of edible insects will continue to rise. Insect larvae can be fed various organic and agricultural by-products, which suggests that insect farming is consistent with the F2F strategy [59]. Use of upcycled organic waste as a substrate for insect farming is a concept of strategic importance because it would help to alleviate the protein shortage in Europe and reduce the volume of agricultural wastes and by-products. Agricultural and food processing wastes and by-products can be effectively upcycled to recover valuable nutrients, and organic waste substrates can be converted into nutritious food products. However, edible insects, as well as other farm animals, are subject to the Feed Ban regulations, which means that use of some by-products in Europe is currently impossible. Insect protein from vertical farms can supplement vegetable protein sources in animal diets and increase availability of farmland for crop production. As a result, edible insect farms can substantially contribute to global food security.

In the EU, processed animal proteins (PAPs) have been approved for use in production of feeds for aquaculture, poultry, pigs, and companion animals [60]. The results of studies and analyses indicate that insect protein is safe for human consumption [61,62,63]. Insect farming is a new agricultural sector, and it can offer new livelihood opportunities for farmers whose livestock has been affected by avian influenza or African swine fever [58]. Insect farming will also contribute to emergence of a new food processing sector, and innovative marketing and production strategies will be required to eliminate negative attitudes to entomophagy and increase popularity of insect-based foods among consumers. As a result, insect farming will create new jobs, promote innovation and enterprise development, and increase food and feed production. Entomophagy is a relatively new concept for European consumers, which is why effective marketing campaigns are needed to increase awareness that insects can be a promising solution to overcoming global food insecurity [64,65,66].

The European Green Deal and the F2F strategy deliver synergistic effects by creating a legislative framework that supports waste recycling and reuse and minimizes the environmental impact of generated waste. In line with these guidelines, PAPs from slaughterhouse wastes should be used in animal nutrition to replace imported soybean meal. As a result, the EU has lifted the 2001 ban on use of PAPs in animal nutrition, excluding PAPs derived from the same species [60]. Use of PAPs of porcine origin is authorized in poultry feed and PAPs of poultry origin in pig feed. These changes should not increase risk of transmission of foodborne pathogens in the food chain. Lifting the species-to-species ban and use of insect protein in livestock nutrition can significantly contribute to development of protein sources alternative to soybean meal, thus improving animal performance and minimizing the environmental impact of livestock production [67]. Therefore, edible insects can be introduced to the human diet both indirectly (through livestock feed) and directly (through consumption) [67,68]. In light of the EU’s agricultural policy, legislative solutions, and future investments in agriculture, edible insects are regarded as a new link in the food chain. However, insect farms should strictly adhere to biosecurity standards, and insect-based foods should be rigorously tested to ensure that foodborne pathogens are not transmitted to consumers.

## 3. The Role of Insects in Spread of Pathogenic Microorganisms and Foodborne Pathogens

Edible insects are regarded as a safe dietary alternative in livestock production [67,68,69]. However, microbiological safety of insect-based foods intended for human consumption is still under debate [29,70]. EFSA outputs on safety evaluation of such products have confirmed safety of edible insect consumption under certain conditions of use [71]. Insect farming can contribute to decreasing prevalence and spread of selected contagious diseases, including foodborne diseases, by eliminating pathogen carriers/reservoirs from the food chain. Due to species specificity and the specific physiology of insects, most entomopathogens do not play a role in epidemiology of zoonoses and do not pose a threat to humans [72]. Arthropods’ ability to transmit foodborne pathogens and vector-borne diseases has been widely researched in the context of food production and the One Health approach [73,74,75]. Edible insects are highly unlikely to act as disease vectors [72,76]. Industrially farmed insects are fed agri-food by-products and plant-based products; therefore, the risk of transmission of zoonotic pathogens is low. Entomopathogens cannot cross the species barrier and cause disease in mammals, which is why edible insects are safe to use in food and feed [72]. It is worth noting that, in some cultures, insects infected with pathogens are regarded as a culinary delicacy or as medicinal products [77,78].

There is no evidence to suggest that edible insects harboring bacterial and viral entomopathogens pose a threat to vertebrates [61,79,80]. However, similar to other foods of animal origin, insect-based foods can raise safety concerns because problems can arise after death of insects and during their processing [81]. Companies that rear and process insects must implement strict sanitary rules to ensure microbiological safety of the end product [10,82,83]. Dedicated processing operations are put into place to eliminate any foodborne pathogens. However, the substrate and end product can become infected during processing. To minimize risk, insect farms should abide by the same biosecurity standards that are applied in the conventional food sector [10,24]. Work surfaces should be disinfected, farm workers should maintain good personal hygiene, farm premises should be regularly cleaned, and safe food preparation and delivery practices should be observed [84]. In farms that have not implemented biosecurity measures, insects and insect-based foods can become contaminated with pathogenic microorganisms transmitted by personnel and pests [53,85]. Therefore, legal regulations, in particular veterinary supervision procedures, should be introduced to guarantee safety of insects as a novel food [86]. Similar to other food products, edible insects are sensitive to deviations from approved production or distribution standards [87,88]. The end product can become contaminated when the required parameters are not observed during acquisition of raw materials, processing (such as drying), transport, storage, and distribution. The associated risks are presented in Table 1. Edible insects as final products should be regularly monitored for presence foodborne pathogens to ensure their safe implementation in the F2F strategy and the European food chain.

Consumption of unprocessed insects may represent a significant risk factor. Insects can act as mechanical or biological vectors of pathogens [73], particularly critical priority pathogens in the food processing industry, including *Bacillus* spp., *Clostridium* spp., *E. coli*, *L. monocytogenes, Salmonella* spp., and *Staphylococcus* spp. [98,99,100,101]. Bacteriological hazards have been most widely investigated, but insects can also act as intermediate hosts or mechanical vectors for parasites in the natural environment [74]. Therefore, effective processing operations should be implemented and sanitary guidelines should be observed to minimize risk of contamination with foodborne pathogens [85].

Allergenicity of edible insects is yet another important safety concern. Similar to other food products, edible insects could pose certain risks to consumers with allergies. To date, 239 arthropod allergens have been identified by the Allergen Nomenclature Sub-committee of the World Health Organization (WHO) [102]. Edible insects may also cause cross-reactivity in people allergic to seafood. The following allergens are most frequently identified in edible insects: fructose-bisphosphate aldolase, phospholipase A, hyaluronidase, arginine kinase, myosin light chain, tropomyosin, α-tubulin, and β-tubulin [103]. A total of 116 allergic reactions to edible insects, mostly grasshoppers, locusts, and lentil weevils, have been identified in 2018 [102]. Insect allergens induce non-specific symptoms, such as anaphylaxis, allergic asthma, hypotension, gastrointestinal symptoms, loss of consciousness, urticaria, erythema, pruritus, and tachycardia. Employees of insect farms and insect processing plants can also develop allergic reactions [104,105]. Allergies also pose a threat to companion animals. Insects can also harbor foreign allergens [103,106], including mites and their metabolites. Direct contact with new proteins or symbiotic organisms can trigger heightened immune response. Presence of gluten in digestive tracts of insects fed grain [107] can pose a threat to people who suffer from celiac disease. Allergizing potential of edible insects should be monitored to eliminate these risks. Potential allergens in insect-based foods should be clearly listed on the product label.

Prions pose a significant biological hazard. Prions are one of the key hazards that have been identified by the European Food Safety Authority (EFSA) in the risk profile of edible insects [61,70]. Insect-specific prion diseases have not been identified because insects lack the gene encoding the prion protein PrP [70,108]. However, insects may act as vectors for prions from contaminated substrates derived from ruminants, which could pose a risk for humans, companion animals, and livestock [61,70].

At present, there is no scientific evidence to suggest that insects pose a viral risk to consumers [61,79,109,110]. Entomoviruses are not pathogenic to humans. Insects are commonly infected with viruses of the family Baculoviridae, which are not dangerous for humans or animals [72,111]. Humans do not harbor insect-specific viruses, and there is negligible risk that new mammalian-specific virus strains will evolve through recombination and reassortment and lead to host switching, as was the case with Swine flu [72]. Edible insects are unlikely to transmit foodborne viruses, such as Hepadnaviridae (hepatitis A and E), Reoviridae (reoviruses), and Caliciviridae (noroviruses) [53]. However, viruses could be transmitted to insects through feed or through contact with farm personnel. Viruses of the family Rhabdoviridae, which cause vesicular stomatitis, have been reported in edible insects [88]. Risk of SARS-CoV-2 transmission by edible insects is very low [72,110,112]. According to Doi et al. [72], risk of infection with SARS-CoV-2 as a foodborne pathogen is negligent in people who consume edible insects [72]. It should be noted that viruses causing foodborne diseases do not replicate in arthropods [79,109], but edible insects could become contaminated during processing and distribution.

Bacteria are presently regarded as the greatest safety hazard in production of edible insects [82]. Due to physiological, environmental, and behavioral differences, every species of edible insects intended for food and feed production harbors different bacteria [100,113]. According to the literature, the microbiome of edible insects poses a negligent risk to consumer safety [114,115]. Several bacteria that can act as opportunist pathogens in humans have been identified in edible insects, but these pathogens are specific to mammals [100]. The risks associated with bacterial symbionts in insects or their potential effects on vertebrates have not been evaluated to date. Insects can act as vectors and carriers of microorganisms that are harmful to humans, particularly when biosecurity and hygiene standards are not observed in insect farms. Insects can carry bacteria that are dangerous to humans, companion animals, and livestock and can act as vectors of foodborne pathogens [116]. Insect microbiota typically include the following bacterial families and genera: Enterobacteriaceae (*Proteus* spp., *Escherichia* spp.), *Pseudomonas* spp., *Staphylococcus* spp., *Streptococcus* spp., *Bacillus* spp., *Micrococcus* spp., *Lactobacillus* spp., and *Acinetobacter* spp. [117]. Some species of the above families and genera are potentially pathogenic to humans, whereas others are commonly encountered in healthy subjects. Unprocessed insects and insect-based foods can harbor *Campylobacter* spp., verotoxic *E. coli*, *Salmonella* spp., and *L. monocytogenes* if microbial inactivation techniques are not applied in production plants. Therefore, insects and insect-based foods should always be screened for these pathogens. Prevalence of some of these pathogens is lower in insects than in other animal protein sources. For example, *Campylobacter* spp. is not replicated in the digestive tract of insects [118,119,120]. Similar risks can be encountered during insect processing. Several bacterial species identified in edible insects can shorten the shelf-life of the final product. Presence of spore-forming bacteria in the end product poses one of the greatest bacteriological hazards [121]. Common sanitation practices, such as drying, boiling, or deep frying, may not be sufficient to eliminate these pathogens.

Entomopathogenic fungi are yet another group of potentially hazardous organisms. There is no scientific evidence to suggest that entomopathogenic fungi pose a risk to vertebrates. In some cultures, these fungi (such as *Ophiocordyceps sinensis*) have long been used in traditional medicine [77]. Mycosporidia could also pose a health threat to consumers [122], but their toxicity has not been analyzed to date. According to the literature, microsporidia *Trachipleistophora* spp. that probably originated from insects can infect vertebrates [123,124]. Due to specific insect rearing conditions and administered feeds, the end product can become contaminated with mycotoxins [125,126]. High concentrations of mycotoxins, such as deoxynivalenol, can lead to gastrointestinal dysfunction in mammals. Molds can also develop in insect-based products that have been stored and distributed in sub-optimal conditions. However, presence of molds in insect-based products has not been reported in the literature. Risks associated with fungi and mycotoxins in insect-derived foods are often disregarded, and further research is needed to guarantee safety of the end product.

Edible insects can potentially transmit parasitic diseases [74,127]. It appears that entomopathogenic parasites are unable to complete their full life cycle in humans or livestock due to biological specificity of the host. Entomopathogenic parasites cannot be transmitted between vertebrates either. However, there is evidence to suggest that some insect-specific parasites can cause digestive problems (such as horsehair worms, *Gordius* spp.) [128] or allergies (*Lophomonas blattarum*) [129]. Insects can also act as intermediate hosts for foodborne pathogens, including tapeworms (*Hymenolepis* spp.), lancet liver flukes (*Dicrocoelium dendriticum*), and nematodes (*Spirocerca lupi*) [127,130,131,132]. Insects can also act as mechanical vectors for different developmental stages of vertebrate parasites in different stages of their life cycle [74,133]. Insects can transmit parasites that colonize body surfaces (hairs, chitin exoskeletons) and digestive tracts. Mechanical transmission of parasites is a serious concern during insect farming. Research has demonstrated that insects can transmit protozoa [127,134,135]. It should also be noted that insects themselves can act as etiological factors of disease. Beetles of the family Tenebrionidae, such as yellow mealworms (*T. molitor*) and lesser mealworms (*A. diaperinus*), can cause canthariasis [136,137,138]. Insect farms can also be colonized by mites [139]. Table 2 provides a summary of potential biological hazards.

## 4. Risk Map

Microbiological safety of edible insects and insect-based foods is currently being extensively researched. The risk that insect-specific pathogens will adapt to new hosts cannot be predicted or ruled out [72]. Foodborne pathogens carried by insects can also pose a threat to immunocompromised and hyper-immunosensitive hosts [141,142]. Therefore, insect-specific microorganisms may turn out to be opportunistic pathogens. The gut microbiome of edible insects is species-specific [100,113], and its impact on mammals suffering from health problems cannot be reliably predicted. New pathogens could also be identified after insect-derived foods have been introduced to the food chain. Employees of livestock farms and food processing farms can be a potential source of infection [84,85,88]. In turn, insect farms require less personnel and can be automated in the future, which will significantly limit risk of pathogen transmission. This threat can be substantially minimized by implementing and rigorously observing biosecurity measures, ISO standards, and hazard analysis and critical control points (HACCP). Sanitary and veterinary supervision measures should also be developed and implemented [84,85,88] in insect farms to reduce risk of pathogen transmission to the level observed in conventional livestock farms. Insects and insect-based foods do not present greater risks than conventional foods because pathogenic microorganisms in both groups of products have low epizootic potential. Only local risks can be anticipated, for example in specific batches of contaminated products [72]. Unlike COVID-19, African swine fever, or avian influenza infections, which are associated with foodborne pathogens and livestock production, there is no evidence to suggest that edible insect farming could contribute to novel pandemic outbreaks [29,72,97,142].

To guarantee the safety of insect-derived foods, edible insects should be reared, processed, and stored according to the same sanitation requirements that are applied in conventional food and feed sectors [82,96]. In view of the biological composition of insect-based products, their microbiological safety, toxicity, palatability, and content of inorganic compounds should be analyzed. The overreaching goal of all processing operations should be to obtain end products that are safe for humans and animals, which can be achieved through implementation of HACCP systems [10,84,85,88]. Quality control measures in insect farms and the hazards associated with edible insects and insect-based foods should be addressed in the HACCP plan [10,84,85,88].

A hypothetical risk map listing the main threats for humans, animals, and insects associated with edible insect farming has been developed based on a review of the literature, the existing knowledge, veterinary regulations, and the authors’ experience. The key risks were represented by groups of biological, chemical, and physical factors. Five groups of biological risk factors, ten groups of chemical risk factors, and thirteen groups of physical risk factors were identified and divided into sub-groups. The risks maps for each category of factors are presented in Supplementary Figures S1–S4. These maps can facilitate identification of the key risks in insect production and choice of the most effective methods for minimizing or eliminating these threats. It should be noted that risk maps present the widest possible range of threats associated with edible insect species classified as novel foods. Individual risk maps should also be developed for each species of edible insects. A combined map of the risks described is included in Supplementary File S1.

Viruses are the first group of biological factors in the risk map. Entomopathogenic viruses belonging to families Baculoviridae (*Granulovirus, Deltabaculovirus*), Iridoviridae (*Iridovirus*), and Reoviridae (*Cypovirus, Dinovernavirus*) pose a potential threat in insect farming. Edible insects can also play a certain role in transmission of pathogenic viruses. Therefore, the following viral families that play an important role in human and veterinary medicine were included in the risk map: Circoviridae, Coronaviridae, Flaviviridae, Herpesviridae, Orthomyxoviridae, Paramyxoviridae, Parvoviridae, and Picornaviridae.

Bacteria constitute the most important group of biological hazards. In the risk map, bacteria were divided into the following groups: symbionts, entomopathogens, and aerobic and anaerobic bacteria that are pathogenic to vertebrates. There is no evidence to suggest that bacterial symbionts in insects pose a health risk for mammals. Bacteria that are pathogenic to insects were divided into two groups: insect-specific (Morganellaceae—*Photorhabdus* spp. and *Xenorhabdus* spp.; Bacillaceae—*Paenibacillus* spp. and *Brevibacillus* spp.) and non-insect-specific pathogens (Pseudomonadaceae—*Pseudomonas* spp.; Streptomycetaceae—*Streptomyces* spp.; Enterobacteriaceae—*Yersinia* spp.). Bacteria that are specific to vertebrates were divided into two groups: anaerobic (Clostridiaceae—*Clostridium* spp.; Campylobacteraceae—*Campylobacter* spp.; Fusobacteriaceae—*Fusobacterium* spp.) and aerobic pathogens (Micrococcaceae—*Micrococcus* spp.; Listeriaceae—*L. monocytogenes*; Enterobacteriaceae—*Enterobacter* spp., *Yersinia* spp., and *Salmonella* spp.). Severity of these biological risks is determined mainly by the type of feed administered to insects.

Fungi are yet another biological risk factor. Inadequate rearing and feed storage conditions can contribute to fungal infections and contamination of the end product. This group of risks includes microsporidia that are pathogenic to mammals (*Encephalitozoon* spp., *Trachipleistophora* spp., and *Tubulinosema* spp.), as well as entomopathogenic microsporidia (*Nosema* spp. and *Paranosema* spp.). Entomopathogenic fungi are also present in the natural environment (Entomophthorales—*Conidiobolus* spp. and *Entomophthora* spp.) and in biological control agents (*Beauveria* spp. and *Metarhizium* spp.). Fungi and mycotoxins that occur commonly in the food chain could also pose biological risks.

Insects play an important role in the life cycle of many pathogens, which is why parasites could also pose a considerable risk in edible insect farming. The following groups of parasites were listed in the risk map: Protozoa, Trematoda, Cestoda, Nematoda, Acanthocephala, and mites. Insects, classified as farmed animals, can become infected with the following entomopathogenic parasites: Sporozoa (*Leidyana* spp., *Gregarine* spp., *Septatorina* spp.), Ciliates (*Tetrahymena* spp., *Nyctotherus* spp.), Nematoda (*Thelastoma* spp., *Steinernema* spp., *Heterorhabditis* spp.), as well as mites, including predatory mites (*Cheyletus eruditus*), opportunistic mites (*Dermatophagoides* spp., *Glicephagus* spp.), and storage mites (*Acarus* spp., *Rhizoglyphus* spp.). All parasites for which insects can act as intermediate or definitive hosts, carriers, mechanical vectors, and reservoirs are potentially harmful to vertebrates.

The last group of biological risk factors are pests that can pose a biosecurity threat in production of edible insects, such as wild animals and other insects. They can carry and transmit pathogens to the farm and lead to contamination of the end product. Attention should also be paid to parasitoids that can spread in the farm environment.

Severity and variation in biological threats are affected by numerous factors, including infectivity and virulence of pathogens, health status of hosts, presence of comorbidities/coinfections, immune status, physiological susceptibility, and history of previous infections. The map of biological risks is largely hypothetical because comprehensive epidemiological and epizootic data are required to fully characterize associated health risks. Edible insects are novel foods, and such detailed information is impossible to acquire at this point. The map of biological risks is presented in Supplementary Figure S1.

Chemical hazards can also be encountered in the edible insect industry [79]. Chemical contaminants can be introduced to insects and end products with the initial stock and feed, as well as by farm employees during biosecurity operations. Some substances can be introduced deliberately (for example, during veterinary treatment) or accidentally (with plant and animal substrates). Chemical substances can be accumulated by insects, which poses a significant threat to consumers. Insect metabolites, such as benzoquinones produced by beetles of the family Tenebrionidae, are also a potential risk factor, which is why stage of insect life cycle is an important consideration. Agri-food by-products can be effectively upcycled in insect rearing, and risk of chemical contamination is also influenced by observance of food safety regulations in crop production and conventional livestock farming. In automated insect rearing and processing systems, technical fluids, such as lubricants, are also a potential source of chemical contamination. Medicinal products used in both human and veterinary medicine, in particular antibiotics, hormones, antiparasitic agents, steroids, sedatives, and analgesics, also pose a risk of chemical contamination in insect farms. Disinfection, disinfestation, and deratting (DDD) measures involving disinfectants, rodenticides, and insecticides carry health risks for vertebrates and reared insects. Insect-based foods can be also contaminated with pesticides, such as acaricides, fungicides, and herbicides. Inadequate storage can lead to spoilage of final products and accumulation of toxic compounds, such as putrescine and indoles. Various chemical substances can be added to insect-derived foods to prevent spoilage, but high concentrations of food preservatives can have toxic effects. Some insects, such as *H. illucens*, can accumulate heavy metals, including arsenic, cadmium, and lead [143], which pose a health threat to consumers. The map of chemical risk factors also includes substances that exert adverse effects on consumers and insects, such as microplastics, bisphenol, and dioxins. Stimulant ingredients in foods, including theophylline, theobromine, nicotine, and caffeine, act as natural insecticides and constitute yet another group of hazardous chemical substances. Effective biosecurity measures should be introduced in insect rearing and processing to prevent or minimize accumulation of external toxins, drugs, and antinutritional factors. The map of chemical risks is presented in Supplementary Figure S2.

The identified physical risks in insect farming are presented in Supplementary Figure S3. Most of these factors do not pose a threat to consumers. Physical hazards, such as fluctuations in humidity and temperature, noise, suboptimal lighting, electromagnetic radiation, and vibration, compromise well-being of insects and influence productivity and profits. However, these factors are not dangerous for consumers. Particulate matter emissions, including PM10 and PM2.5, during insect rearing and processing can cause allergies in consumers and farm employees. Chitin can lead to gastrointestinal tract irritation in humans and animals. Moreover, dust containing chitin particles may pose a risk of airway irritation in farms where insects are fed agri-food by-products: there is a risk that the end product will contain hard particles (plant and animal residues, soil, or gravel). Therefore, insects and feed should be thoroughly cleaned before being converted into food products. Similar to conventional foods, insect-based products can also be contaminated with microplastics and micrometals that pose a threat to humans and animals.

## 5. Safety of Insects Reared for Food and Feed

Processed insects and insect-based foods have to adhere to food safety standards set forth by legal regulations [10,25,61,86,144]. Legal provisions play a key role in production and marketing of insect-based foods. Insect species that can be included in formulation of food and feed products have been listed in Commission Regulation (EU) 2017/893 of 24 May 2017 Amending Annexes I and IV to Regulation (EC) No. 999/2001 of the European Parliament and of the Council and Annexes X, XIV, and XV to Commission Regulation (EU) No. 142/2011 as Regards the Provisions on Processed Animal Protein [145]. In the EU, the risk profile and potential hazards associated with farmed insects used as food and feed were described in the EFSA opinion of 8 October 2015 [61,62,63]. The identified insect species do not transmit pathogens specific to plants and vertebrates. These insects are not invasive or pathogenic to mammals; they do not exert a negative impact on crops, and they are not protected [10]. It should also be noted that both whole edible insects and insect-based foods can be introduced to the EU market [23]. Similar to conventional livestock, edible insects have to be monitored to ensure the safety of the produced food and feed. Most legal regulations concern hygiene standards in food and feed production (Commission Regulation (EC) No. 1069/2009; Commission Regulation (EU) No. 142/2011; Commission Implementing Regulation (EU) 2017/2469) [23,146,147]. Observance of safety standards in the food processing sector is monitored by the respective authorities, including veterinary and sanitary inspectorates.

To eliminate foodborne pathogens, insect-based foods placed on the market must meet food and feed hygiene standards, good breeding practices, good hygiene practices, and good production practices [24,69,113,148,149]. Edible insects are classified as farmed animals; therefore, they can only be fed plant- and animal-based materials that have been approved for livestock nutrition [10]. Materials acquired outside the food chain may not be used as feed in insect farms. To minimize transmission of foodborne pathogens, commercial insect feeds must be purchased from certified manufacturers who adhere to HACCP requirements and European feed laws [150,151]. Insect producers must keep documents that confirm feed delivery dates and list feed manufacturers and initial feed parameters [150,151]. Products that do not meet safety standards (moldy feeds, feeds withdrawn from the market) cannot be fed to insects or processed into feed [150,152]. Each batch of insects placed on the food and feed market must conform to microbiological safety standards and maximum residue limits (MRL) stipulated in the relevant regulations [88]. Insects should be regularly monitored for presence of undesirable chemical substances, such as heavy metals, pesticides, and mycotoxins [85]. Each product batch should be clearly marked in every stage of the production process to ensure food traceability [10,152].

Applicability of animal-based substrates as insect feeds and the relevant processing requirements are set forth by Commission Notice (2018/C 133/02)—Guidelines for the Feed Use of Food No Longer Intended for Human Consumption [153]. Most farmed insects will be processed into feed, which is why contamination with toxic compounds is a valid concern. Due to their specific physiology, edible insects can accumulate heavy metals [125,143]. Heavy metals have been identified in black soldier fly prepupae [143,154]. Microbiological contamination poses yet another threat. Insect farming conditions and food sources can promote development of specific pathogens. Some insects are capable of reducing microbial counts in digested food, but risk of microbiological contamination cannot be ruled out. For example, the black soldier fly can reduce microbial counts in alkaline poultry excreta but not in acidic pig manure [155,156,157]. In addition, not all pathogens (such as parasitic eggs) are effectively eliminated by the black soldier fly [158]. Effective treatments, such as high-temperature processing, are needed to minimize counts of pathogenic microorganisms in farmed insects [82,83,159]. Such treatments eliminate foodborne pathogens and microorganisms that cause food spoilage. Both insect feeds and end products must be free of pesticides, antibiotics, detergents, and other contaminants [150].

The described hazards and associated adverse health effects should be considered in qualitative and quantitative risk analyses [53]. Various microbiological hazards are associated with presence of pathogenic bacteria, such as *Campylobacter* spp.*, S. aureus, B. cereus, E. coli, C. perfringens,* and *Enterococcus* spp. They should be monitored in production of insect-based foods, even if the relevant limits have not yet been introduced in the insect sector [53]. Samples of the end product have to meet guideline microbiological limits for *Salmonella* spp. (not detected in 25 g) and Enterobacteriaceae (up to 300 CFU in 1 g). Food products listed in Annex IV to Commission Regulation (EU) No. 142/2011 must be free of *C. perfringens* (1 g samples) [147]. According to Commission Regulation (EC) No. 2073/2005, *L. monocytogenes* counts in ready-to-eat foods may not exceed 100 CFU per 1 g of the product [160]. The above regulation also introduced microbiological limits for raw materials, minced meat and meat products (absence of *Salmonella* spp. in 10 g of minced meat and meat preparations that are made from species other than poultry and are intended to be eaten cooked; *E. coli*—up to 500 CFU/g in minced meat at the end of the manufacturing process), and cooked crustaceans and molluscan shellfish (absence of *Salmonella* spp. in 25 g of the product). If required, insects should also be periodically inspected for other pathogens and chemical substances, including pesticides, heavy metals, dioxins, and mycotoxins (Directive 2002/32/EC of the European Parliament and of the Council of 7 May 2002 on undesirable substances in animal feed). Insects should also be analyzed for presence of physical contaminants, such as plastic and metal components, and foreign particles [150], as well as physical parameters, such as water activity [61,94].

## 6. Conclusions

Insects have been long reared and consumed in many regions of the world (such as Southeast Asia, Mexico, and Africa), but little is known about their ability to transmit foodborne pathogens and their safety for consumers. In Europe, consumer attitudes toward entomophagy are gradually changing, and both whole insects and insect-based foods are gaining popularity. Therefore, food safety standards and veterinary inspection procedures targeting insect farms will have to be implemented to guarantee safety of European consumers. Despite the fact that the edible insect industry is a completely new sector of European agriculture, it will contribute to achievement of the main goals of the F2F strategy, which lies at the heart of the European Green Deal. The European Green Deal proposes a sustainable and inclusive growth strategy to improve consumers’ health, care for the environment, and leave no one behind. The European Food Safety Authority has initiated a debate on strategic importance of edible insects for the European food and feed market (evaluation of insect-based foods, authorization of insect protein in poultry and pig feed). The F2F strategy and the resulting reforms in the EU’s agricultural policy are major milestones on the path to a more sustainable food supply chain. Edible insects have been classified as farmed animals, but little remains known about their biology, physiology, biochemical pathways, specific pathogens, treatment options, and humanitarian rearing methods. Insect welfare and ethical criteria in insect farming are difficult to define. Veterinarians, in their daily practice, deal with insects, including pests or ectoparasites and bees, but edible insects are new and enigmatic because they have to compare them to conventional livestock. Action should be taken to educate veterinarians about farmed insects. Even though insects constitute a novel link in the food chain, scientists, veterinary practitioners, and breeders must face and solve the same old problems that are encountered in conventional livestock farming and food production.

## Figures and Tables

**Table 1:** Possible routes of contamination of edible insects and insect-based foods.

Stages of Contamination	Risks	Treatment	Reference
Substrate	(Crickets) Minimal impact of external microbiota. (Crickets) Bacterial endospore counts in crickets fed a standard + farm weed (S + W) diet were significantly lower and thus promising and could reduce risks associated with ready-to-eat insects. Risk of contamination with *Salmonella* spp. and *Campylobacter* spp. increases if materials such as used paper egg cartons are utilized in insect rearing. This risk is higher if cartons had been in contact with poultry feces.		[89,90,91]
Rearing	(Crickets) *Aspergillus flavus* strains with low mycotoxigenic potential were identified in reared crickets, which could point to presence of mycotoxins in edible crickets.		[89]
Harvest	(Crickets) Starvation is not an effective method for reducing microbial loads in edible crickets.	Gut emptying by starvation prior to killing could reduce the microbial load in the insect gut, but it could also decrease fat and energy content and profitability of production.	[92]
Processing	(Crickets) High microbial loads of TAC and Enterobacteriaceae were detected in edible crickets, indicating a high risk of rapid spoilage. (Crickets) Sporulating bacteria are a part of the cricket microbiome Food safety risks associated with viruses are very low. *Vibrio* spp., *Streptococcus* spp., *Staphylococcus* spp., *Clostridium* spp., and *Bacillus* spp. were identified in several studies on the microbiota of processed edible insects sold online.	Thermal treatments, novel processing methods (i.e., high-pressure processing), and additional post-processing treatments (acidification, addition of food preservatives, modified atmosphere packaging, etc.) should be applied to extend crickets’ shelf-life.	[89,93,94]
Transport		https://ipiff.org/wp-content/uploads/2019/12/IPIFF-Guide-on-Good-Hygiene-Practices.pdf (accessed on: 13.November.2022)	
Preparation	Dried mopane worms, termites, and stink bugs sold at the Thohoyandou market were characterized by low contamination with coliforms, *Escherichia coli, Staphylococcus aureus*, *Salmonella* spp., TPC, yeasts, and molds.		[95]
Storage	(*T. molitor, Alphitobius diaperinus, Gryllus assimilis, Lo. Migratoria*) microbiological characteristics in different storage periods—safe for human consumption.	Insects intended for long-term storage should be killed in boiling water, dried at 103 °C for 12 h, and hermetically packed.	[96]
Consumption	The nutritional value and the microbiological and toxicological profiles of insects are influenced by composition of organic side streams. The microbial risks associated with edible insects can be substantially reduced by observing good hygienic practices in rearing, handling, harvesting, processing, storage, and transport of insects and insect-based products. Several spoilage-causing microbes that can alter food quality, including *Lysinibacillus* sp. and *Bacillus subtilis*, have been detected in edible insects. Yeasts, including *Tetrapisispora* spp*., Candida* spp*., Pichia* spp., and *Debaryomyces* spp*.,* and molds, including *Aspergillus* spp*., Alternaria* spp*., Cladosporium* spp*., Fusarium* spp*., Penicillium* spp*., Phycomycetes* spp*.,* and *Wallemia* spp., are associated with the microbiota found on the body surface or in the gut of edible insects and may be harmful. 38 samples of deep-fried and spiced *Ach. Domesticus*, *Lo. Migratoria*, and *Omphisa fuscidentalis* tested negative for *Salmonella* spp., *Listeria monocytogenes, E. coli*, and *S aureus*, but dried and powdered insects, as well as pollen, contained *Bacillus cereus*, coliforms, *Serratia liquefaciens, Listeria ivanovii, Mucor* spp., A*spergillus* spp., *Penicillium* spp., and *Cryptococcus neoformans.*		[18,28,93,97]
R&D	(Crickets) Further efforts are needed to identify food-borne pathogens in edible crickets and define possible bacterial quality reference values.		[89]

**Table 2:** Biological hazards associated with different species of edible insects.

Type of Hazard	Infectious Agent	Sensitive Species	Predisposing Factors	References
Prion vectors	Proteinaceous infectious particles	All species fed contaminated substrates of animal origin	inadequate rearing practices failure to observe legal regulations contaminated feed and litter handling operations absence of biosecurity measures sanitation requirements are not observed by farm personnel	[70,108]
Viruses	Caliciviridae Hepadnaviridae Vesicular stomatitis virus (VSV)	Migratory locust (*Lo. migratoria*), black soldier fly (*H. illucens*) Insects harvested from the natural environment	insects are reared with other animals absence of biosecurity measures sanitation requirements are not observed	[53,88]
Bacteria	*Aeromonas hydrophila*, *B. cereus*, *Clostridium difficile*, *Clostridium perfringens*, *Clostridium septicum*, *Clostridium sporogenes*, *E. coli*, *Enterococcus faecium*, *Enterococcus faecalis*, *Listeria* spp., *Salmonella* spp., *S. aureu*s.	Migratory locust (*Lo. migratoria*) Yellow mealworm (*T. molitor*) Lesser mealworm (*A. diaperinus*) House cricket (*Ach. domesticus*) Domestic silk moth (*B. mori*) Insects harvested from the natural environment	handling operations deviations from production standards rearing conditions inadequate rearing practices contamination of feed and litter	[98,99,100,101,117]
Fungi and mycotoxins	*Aspergillus fumigatus*, *Aspergillus sclerotiorum*, *Cladosporium* spp. *Penicillium* spp., *Fusarium* spp., *Phycomycetes* spp. Microsporidia	Migratory locust (*Lo. migratoria*) Black soldier fly (*H. illucens*) Yellow mealworm (*T. molitor*)	high humidity contamination of feed and litter high water activity in the end product inadequate storage conditions	[28,83,125,140]
Parasites	Protozoa (*Balantidium* spp., *Cryptosporidium* spp., *Entamoeba* spp.) Trematoda (*Dicrocoelium* spp., Lecithodendriidae) Cestoda (*Hymenolepis* spp., *Raillietina* spp.) Nematoda (*Gordius* spp., *Spirocerca* spp.)	Yellow mealworm (*T. molitor*) Lesser mealworm (*A. diaperinus*) House cricket (*Ach domesticus*) Insects harvested from the natural environment	insects as vectors of parasitic infections insects as intermediate hosts insects harvested in the natural environment absence of biosecurity measures dirty and contaminated feed (such as unwashed vegetables) presence of pests farm/processing personnel do not observe sanitation requirements insects are reared with other animals	[4,127,128,129,130,131,132,133,134,135]
Mites	*Acarus* spp., *Dermatophagoides* spp., *Goheria* spp. *Tyrophagus* spp.	Mealworm (*T molitor*) Lesser mealworm (*A. diaperinus*) Black soldier fly (*H. illucens*) House cricket (*Ach. domesticus*)	feed substrates are contaminated with mites in different stages of the life cycle biosecurity measures are not observed sanitation requirements are not observed high humidity residual feed is not removed from farm premises	[139]

## Data Availability

Data are contained within the article.

## Supplementary Materials

The following supporting information can be downloaded at: https://www.mdpi.com/article/10.3390/foods12040770/s1, Figure S1: Map of biological risk factors that pose a potential threat to vertebrates and edible insects. Figure S2: Map of chemical risk factors that pose a potential threat to vertebrates and edible insects. Figure S3: Map of physical risk factors that pose a potential threat to vertebrates and edible insects. Figure S4: Risk map for insect breeding, insect processing, and insect-based food and feed.
