# Supplementary figures and images for: Foodborne Diseases in the Edible Insect Industry in Europe—New Challenges and Old Problems
Source: Foods. 2023 Feb 10;12(4):770. doi: 10.3390/foods12040770 (PMC9956073; doi:10.3390/foods12040770)

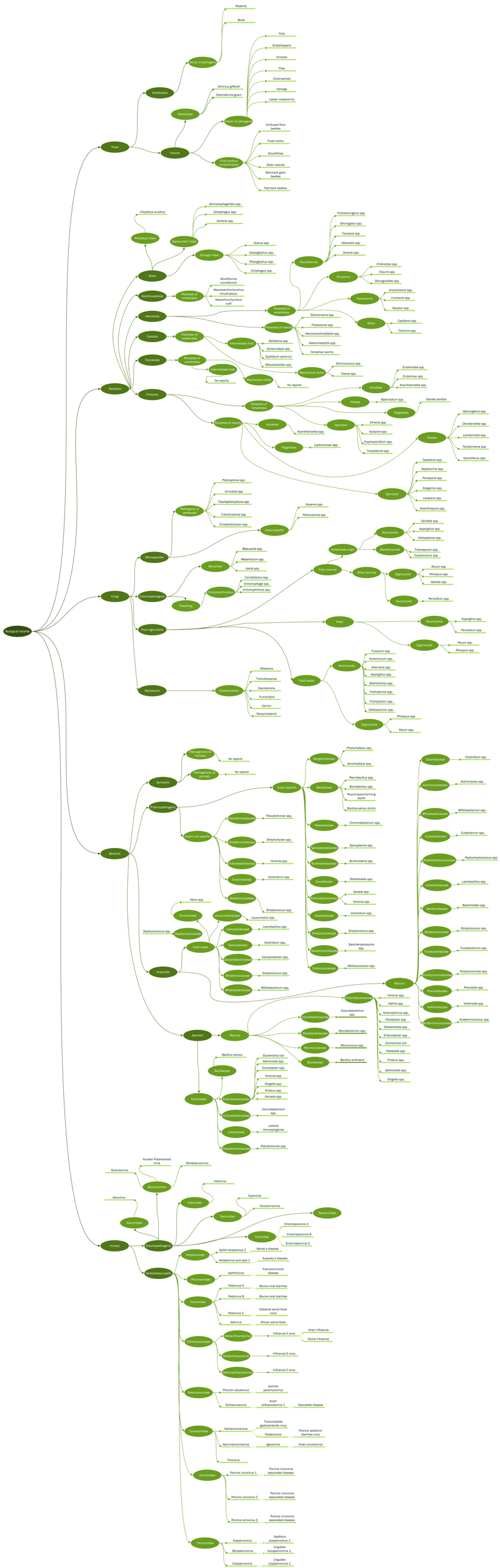

Supplement: Supplementary file 1 [file foods-12-00770-s001.zip › Supplementary Figure S1.pdf]

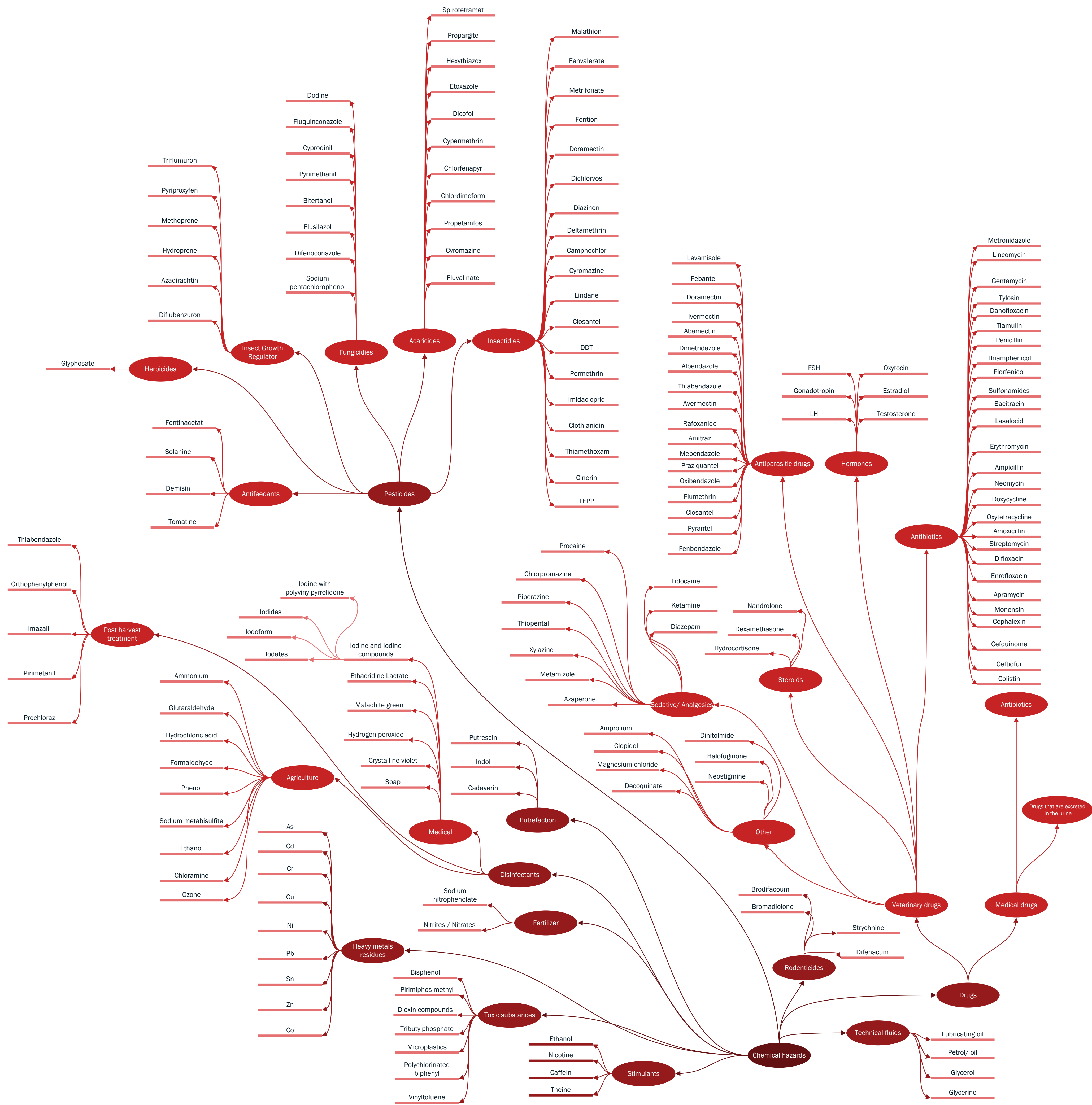

Supplement: Supplementary file 1 [file foods-12-00770-s001.zip › Supplementary Figure S2.pdf]

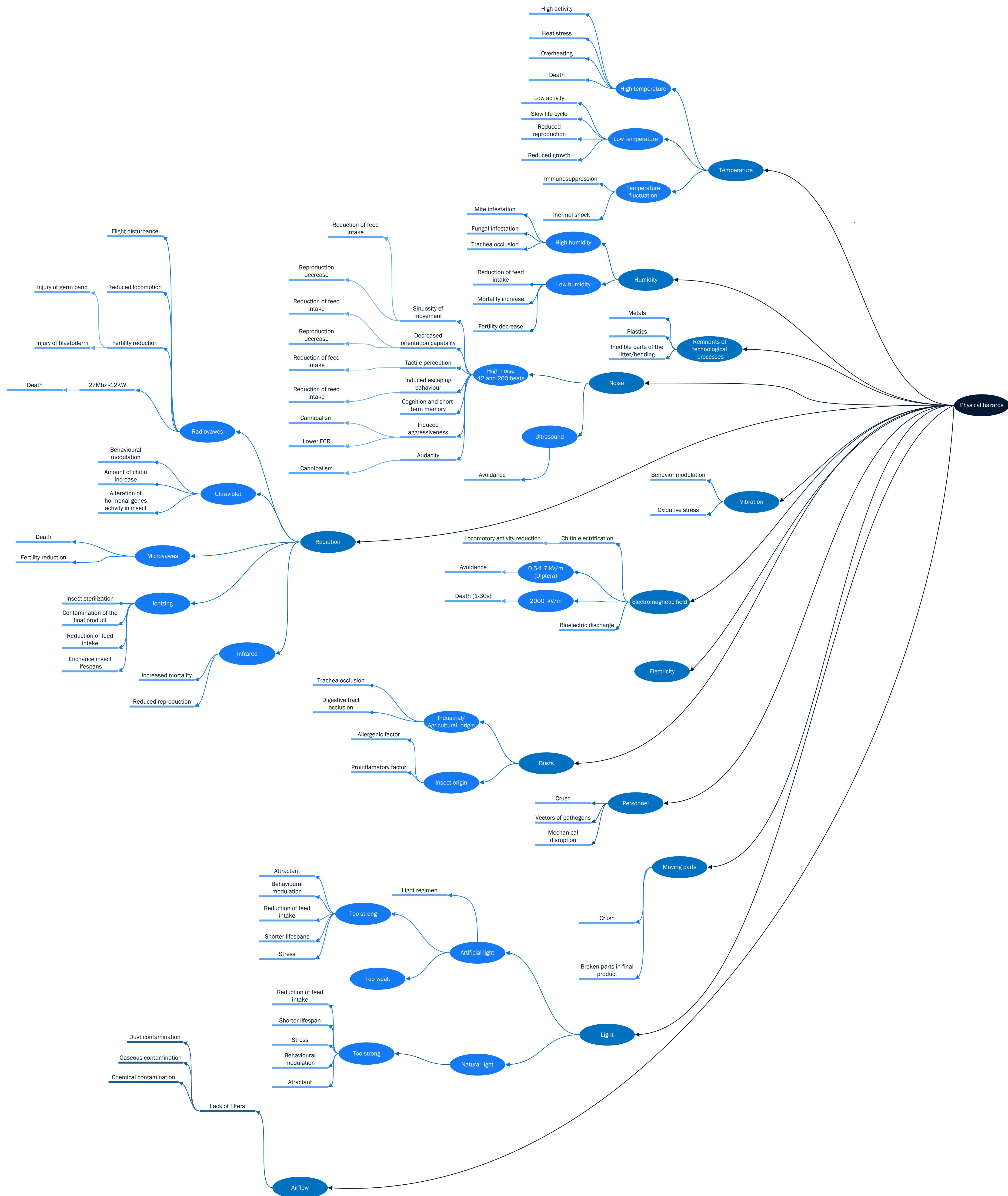

Supplement: Supplementary file 1 [file foods-12-00770-s001.zip › Supplementary Figure S3.pdf]

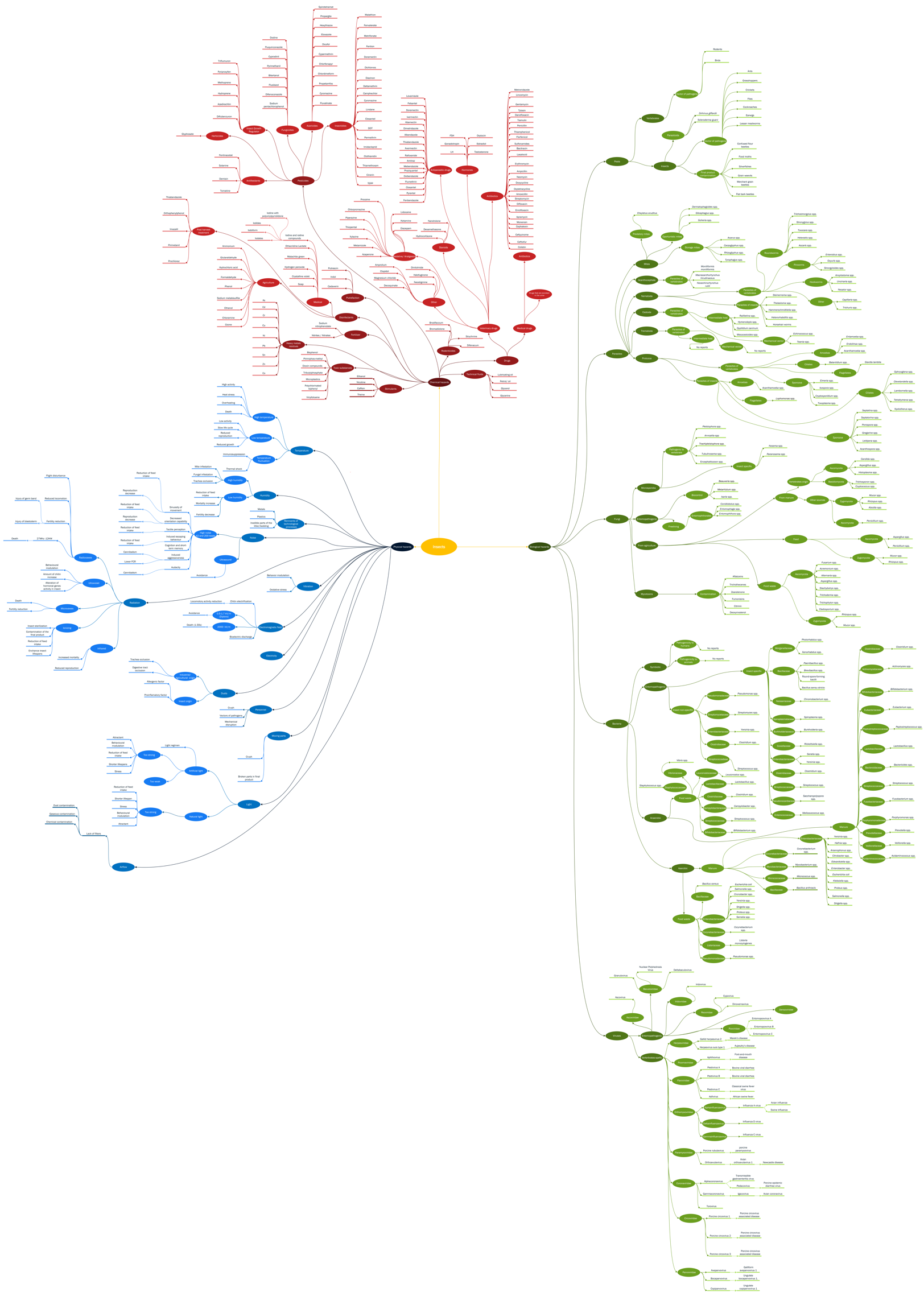

Supplement: Supplementary file 1 [file foods-12-00770-s001.zip › Supplementary Figure S4.pdf]
